# Calculating Super Efficiency of DMUs for Ranking Units in Data Envelopment Analysis Based on SBM Model

**DOI:** 10.1155/2014/382390

**Published:** 2014-08-19

**Authors:** E. Zanboori, M. Rostamy-Malkhalifeh, G. R. Jahanshahloo, N. Shoja

**Affiliations:** ^1^Department of Mathematics, Islamic Azad University, Science and Research Branch, Tehran, Iran; ^2^Department of Mathematics, Islamic Azad University, Firozkoh Branch, Firozkoh, Iran

## Abstract

There are a number of methods for ranking decision making units (DMUs), among which calculating super efficiency and then ranking the units based on the obtained amount of super efficiency are both valid and efficient. Since most of the proposed models do not provide the projection of Pareto efficiency, a model is developed and presented through this paper based on which in the projection of Pareto-efficient is obtained, in addition to calculating the amount of super efficiency. Moreover, the model is unit invariant, and is always feasible and makes the amount of inefficiency effective in ranking.

## 1. Introduction

Since in the current world a wide variety of companies and organizations of different areas work in common ground under the supervision of a common manager (like central bank in a country or other banks and respective branches), in order to improve the performance of units, a manager in addition to evaluation should rank them and present an efficient pattern corresponding to inefficient units. Data envelopment analysis (DEA) is a technique for calculating the amount of efficiency in DMUs which have multiple inputs and outputs. According to the obtained efficiency through this technique, these units can be ranked and distinction can be made between efficient units and inefficient ones [1–4].

So far, a number of studies have been conducted for ranking DMUs and according to them various models have been proposed. Banker et al. in [5] using additive model evaluated DMUs and ranked them. Sexton's cross-efficiency method is another method of ranking presented in 1986 [6]. In this method, first, all DMUs are assessed by multiplier model and then optimal weights corresponding to each unit are considered for other units and the amount of objective function is measured. Afterwards, for each unit, the obtained amounts are combined through averaging and, by considering the achieved number, DMUs are ranked. The disadvantages of this method can be attributed to the presence of multiple optimal solutions as well as unreliability of averaging in unit rank. It is worth mentioning that many researchers such as [7–13] have proposed different models for improving sexton method. Andersen and Petersen in 1993 proposed a method in which they could rank extreme efficient units by eliminating unit under evaluation of production possibility set (PPS) and forming a new PPS [14]. In 1998, Mehrabian et al. using weight constraints on input and output weights in A.P. model solved some of its problems such as instability; however, others including the ranking of nonextreme efficient units, lack of presenting Pareto-efficient projection, and infeasibility in some cases still remained [15]. Li et al. (1999) modified Mehrabian et al.'s model and simultaneously by enhancing outputs and reducing inputs to the same extent resolved the mentioned infeasibility [16]. Sueyoshi (1999) adding weight constraints to CCR multiplier model developed an approach named benchmarking method. Their model like A.P. suffered infeasibility in some cases. Sueyoshi introduced AIN parameter for the purpose of ranking extreme efficient units [6]. Common weight is another method developed in 2000 by Hosseinzadeh Lotfi et al. for ranking units. That model through which units were evaluated and ranked was multiobjective; however, after specific transformation, a nonlinear programming model resulted [17]. In 2004, gradient line method was introduced by Jahanshahloo et al. for ranking extreme efficient units [18]. This method was always feasible despite the fact that it does not provide any suggestion for ranking the nonextreme efficient units. Jahanshahloo et al. presented other methods such as Mont Carlo, norms (*L*_1_ and *L*_*∞*_), Chebyshev norm, and concept of advantage, all of which rank the units in a way [4, 19–27]. In addition, there exist some other ranking methods not much developed and extended in the literature [4, 7, 18, 20, 21, 28–33].

Among the above-mentioned methods, A.P. is the one which has been mostly used despite its disadvantages like lack of finding Pareto-efficient projection, infeasible cases, lack of ranking nonextreme efficient units, and finally lack of stability corresponding to data transformation. A number of researchers have proposed various models and attempted to modify it and eliminate its problems [14, 16, 27, 34–45].

In this paper, besides ranking DMUs, a projection of Pareto-efficient is presented. Also, the amount of inefficiency has been involved in the ranking. The model is always feasible and stable corresponding to data transformation. In this paper, first SBM model and then Tone super efficiency are presented. Afterwards, the proposed model along with some theorems is introduced. Then, using 2 different numerical examples, the proposed model is compared with Tone model and the results are revealed.

## 2. Preliminaries

Suppose that there is a set of *n* DMUs {DMU_*j*_ : *j* = 1,2,…, *n*}, producing *s* outputs *y*_*rj*_(*r* = 1,2,…, *s*) by consuming *m* inputs. Assume that all the vector of *x*_*ij*_(*i* = 1,…, *m*) inputs and outputs are not negative and are opposed to zero. The production possibility set (PPS) spanned by all DMUs is defined as follows:

(1) P={(x1,…,xm,y1,…,ys) ∣ xi≥∑j=1nλjxij,i=1,…,m,  yr≤∑j=1nλjyrj,r=1,…,s,λj≥0}.

Based upon the constant return to scale (CRS), the SBM model is described as follows. It assumed that *x*_*ij*_ > 0(*i* = 1,…, *m*,  *j* = 1,2,…, *n*) and *y*_*rj*_ > 0  (*r* = 1,2,…, *s*,  *j* = 1,2,…, *n*):

(2) Min: ρ=1−(1/m)∑i=1m(zi−/xik)1+(1/s)∑r=1s(zr+/yrk)s.t.:  ∑j=1nλjxij+zi−=xik, i=1,…,m,   ∑j=1nλjyrj−zr+=  yrk, r=1,…,s,      λj≥0, zr≥0, zi≥0.

**Definition 1 .**

DMU_*k*_ in model (2) is defined as an efficient unit if and only if ρ* = 1. In other words, DMU_*k*_ is SBM-efficient, whenever *z*_*i*_^∗−^ = 0, *z*_*i*_^∗+^ = 0.

In order to define super efficiency model corresponding to model (2), to obtain new PPS without considering  DMU_*k*_, *P*_*c*_′ is as the following:

(3) Pc′={(x1,…,xm,y1,…,ys) ∣ x≥∑j=1,j≠knλjxj,   y≤∑j=1,j≠knλjyj,λj≥0,j≠k}.

Tone introduced model (4) for calculating super efficiency of DMUs based on production possibility set *P*_*c*_′:

(4) Min: δ=(1/m)∑i=1m(x−i/xik)(1/s)∑r=1s(y−r/yrk)s.t.:  ∑j=1,j≠knλjxij≤x−i, i=1,…,m,   ∑j=1,j≠knλjyrj≥  y−r, r=1,…,s,    λj≥0, j=1,…,n,  j≠k,        x−i≥xik, i =1,…,m,   y−r≥0, y−r≤yrk, r=1,…,s.

If nonefficient units were evaluated through the above model, the efficient score for all of them would be equal to one. In other words, nonefficient units could not be distinguished. Therefore, both efficient and nonefficient units should be considered discretely and it firstly requires solving SBM model for all DMUs and then distinguishing efficient units from nonefficient ones and, finally, super efficiency score is obtained.

This paper through the coming sections seeks to present a model based on which super efficiency score for nonefficient units is measured and projection of Pareto-efficient for all units is obtained.

## 3. Proposed Model

In this section, a model is introduced which its notion is based on the minimum distance from nonradial view. In this model, using the fact that input vector (output vector) corresponding to each unit is not exactly equal to zero, the maximum amount of each component is obtained from input and output vectors. Then, considering these amounts and also PPS of *P*_*c*_′, all units are solved by model (5). In this model, the point from *P*_*c*_′ frontier to the unit under evaluation is obtained as follows:

(5) Min: 1+(1/m)∑i=1m(tiximax⁡/xik)1−(1/s)∑r=1s(tryrmax⁡/yrk)s.t.:   ∑j=1,j≠knλjxij−ti−ximax⁡≤xik, ∀i,   ∑j=1,j≠knλjyrj+tr+yrmax⁡≥  yrk, ∀r,        λj≥0, ti−≥0, tr+≥0.

**Figure 1:**
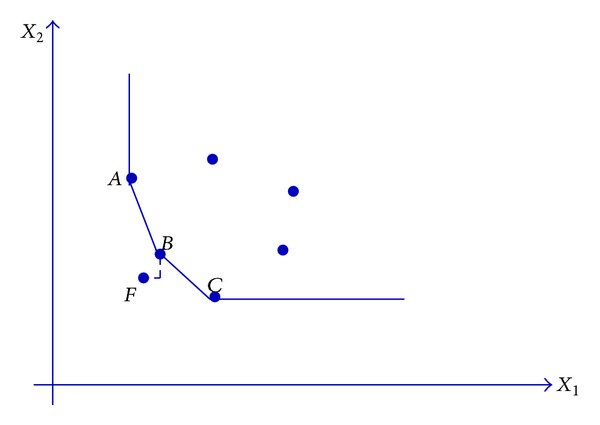


**Figure 2:**
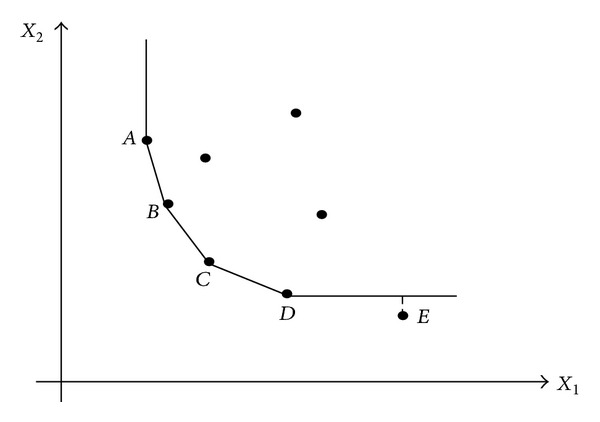


The point (*x*_*ik*_ + *t*_*i*_^−∗^ *x*_*i*_^max⁡^, *y*_*rk*_ − *t*_*r*_^+∗^ *y*_*r*_^max⁡^) is the point of *P*_*c*_′. Regarding the position of units in *P*_*c*_′ and the obtained projection of the unit under evaluation by model (5), two cases are probable to happen. The first case is like DMU_*f*_  of Figure 1 in which the obtained projection is placed on the strong frontier while the second case is similar to DMU_*e*_ in Figure 2 in which its projection is on the weak frontier.

Since projection point may lie on weak frontier, for the purpose of finding Pareto-efficient projection point for all units under evaluation, model (6) should be solved. In this model, (*t*_*i*_^−∗^ *x*_*i*_^max⁡^, *t*_*r*_^+∗^ *y*_*r*_^max⁡^) is the optimal solution of model (5):

(6) Min:   1−(1/m)∑i=1m(si−/xik)1+(1/s)∑r=1s(sr+/yrk)s.t.:   ∑j=1,j≠knλjxij−t  i−∗ximax⁡+si−=xik, ∀i,    ∑j=1,j≠knλjyrj+tr+∗yrmax⁡−sr+=  yrk, ∀r,          λj≥0, sr≥0, si≥0.

Through this, first by adding input saving (*t*_*i*_^−∗^ *x*_*i*_^max⁡^) and subtracting output surpluses (*t*_*r*_^+∗^ *y*_*r*_^max⁡^) to and from the unit under evaluation, it moves to a point of *P*_*c*_′ which is a frontier point. Moreover, since it may lie on the weak frontier, it is projected on Pareto-efficient point by using model (6).

In this way, if DMU is nonefficient, *t*_*i*_^−∗^ = *t*_*r*_^+∗^ = 0. Consequently, for its projection model (6) which is in fact the same as SBM model is used. Super efficiency score in this method for  DMU_*k*_  is defined as follows:

(∗) $$\begin{array}{c} \varphi^{*}=\{\begin{array}{cc} \frac{1+\left(1/m\right)\sum_{i=1}^{m}\left(\left(t_{i}^{-*}x_{i}^{\max}-s_{i}^{*-}\right)/x_{ik}\right)}{1-\left(1/s\right)\sum_{r=1}^{s}\left(\left(t_{r}^{+*}y_{r}^{\max}-s_{r}^{*+}\right)/y_{rk}\right)}\;\;\;\; & \\ \;\;\text{if}\;\;\delta^{*}=\frac{1+\left(1/m\right)\sum_{i=1}^{m}\left(t_{i}^{-*}x_{i}^{\max}/x_{ik}\right)}{1-\left(1/s\right)\sum_{r=1}^{s}\left(t_{r}^{+*}y_{r}^{\max}/y_{rk}\right)}>1 & \\ \frac{1-\left(1/m\right)\sum_{i=1}^{m}\left(s_{i}^{*-}/x_{ik}\right)}{1+\left(1/s\right)\sum_{r=1}^{s}\left(s_{r}^{*+}/y_{rk}\right)}\;\;\;\;\;\;\;\; & \\ \;\;\text{Otherwise}.\;\;\;\;\;\;\;\;\;\;\;\;\; & \end{array} \end{array}$$

As it is observed in the above definition, if the first projection of the unit under evaluation like  DMU_*e*_  lies on the weak frontier, the second projection which is Pareto-efficient is considered for this unit. Furthermore, the amount of slack variables (*s*_*i*_^−^, *s*_*r*_^+^) is included in the definition.

**Theorem 2 .**

Model (4) and model (5) are equivalent.

**Proof**

As can be seen in model (2), ${\overset{-}{x}}_{i}\geq x_{ik}$,  ${\overset{-}{y}}_{r}\leq y_{rk}$. Substitute  ${\overset{-}{x}}_{i}=x_{ik}+t_{i}^{-}x_{i}^{\max}$ and ${\overset{-}{y}}_{r}=y_{rk}+t_{r}^{+}y_{r}^{\max}$ in model (4) and rewrite the following:

(7) Min: δ=(1/m)∑i=1m((xik+ti−ximax⁡)/xik)(1/s)∑r=1s((yrk−tr+yrmax⁡)/yrk)s.t.:  ∑j=1,j≠knλjxij≤xik+ti−ximax⁡, i=1,…,m,   ∑j=1,j≠knλjyrj≥yrk+tr+yrmax⁡, r=1,…,s,        λj≥0, j=1,…,n, j≠k,           ti−≥0, i=1,…,m,            tr+≥0, r=1,…,s.

The solution space of both model (6) and model (7) is equal. After rearrangement, simplifying the objective function of model (7), we will have the following:

(8) Min: δ=(1/m)∑i=1m((xik+ti−ximax⁡)/xik)(1/s)∑r=1s((yrk−tr+yrmax⁡)/yrk)    =1+(1/m)∑i=1m(ti−ximax⁡/xik)1−(1/s)∑r=1s(tr+yrmax⁡/yrk)s.t.:  ∑j=1,j≠knλjxij−ti−ximax⁡≤xik, i=1,…,m,   ∑j=1,j≠knλjyrj+tr+yrmax⁡≥yrk, r=1,…,s,        λj≥0, j=1,…,n, j≠k,             ti−≥0, i=1,…,m,            tr+≥0, r=1,…,s.

It can be seen that model (8) is the same as model (5).

**Theorem 3 .**

If *DMU*_*k*_ ∉ *P*_*c*_′, φ* ≤ δ*.

**Proof**

Since DMU_*k*_ ∉ *P*_*c*_′, there exists an *i* in model (5), in which *t*_*i*_^−∗^ > 0 or there exists an *r* in which *t*_*r*_^+∗^ > 0. Thus  (1 + (1/*m*)∑_*i*=1_^*m*^(*t*_*i*_^−∗^ *x*_*i*_^max⁡^/*x*_*ik*_))/(1 − (1/*s*)∑_*r*=1_^*s*^(*t*_*r*_^+∗^ *y*_*r*_^max⁡^/*y*_*rk*_)) > 1. Depending on the position of projection point of DMU_*k*_ on strong frontier or weak frontier, the amounts of *s*_*i*_^∗−^, *s*_*r*_^∗+^ are greater than or equal to zero in the definition of φ*. Therefore, φ* = (1 + (1/*m*)∑_*i*=1_^*m*^((*t*_*i*_^−∗^ *x*_*i*_^max⁡^ − *s*_*i*_^∗−^)/*x*_*ik*_))/(1 − (1/*s*)∑_*r*=1_^*s*^((*t*_*r*_^+∗^ *y*_*r*_^max⁡^ − *s*_*r*_^∗+^)/*y*_*rk*_)) ≤ (1 + (1/*m*)∑_*i*=1_^*m*^(*t*_*i*_^−∗^ *x*_*i*_^max⁡^/*x*_*ik*_))/(1 − (1/*s*)∑_*r*=1_^*s*^(*t*_*r*_^+∗^ *y*_*r*_^max⁡^/*y*_*rk*_)) = δ*.

**Theorem 4 .**

If *DMU*_*k*_ belongs to *P*_*c*_′, φ* = ρ*.

**Proof**

Since DMU_*k*_ ∈ *P*_*c*_′, using model (5), *t*_*i*_^−∗^ = *t*_*r*_^+∗^ = 0. Then, substituting them in model (6), model (2) is obtained and also *s*_*i*_^∗−^ = *z*_*i*_^∗−^,  *s*_*r*_^∗+^ = *z*_*r*_^∗+^. Therefore, by definition of super efficiency, *(∗)*: φ* = (1 − (1/*m*)∑_*i*=1_^*m*^(*s*_*i*_^∗−^/*x*_*ik*_))/(1 + (1/*s*)∑_*r*=1_^*s*^(*s*_*r*_^∗+^/*y*_*rk*_)) = (1 − (1/*m*)∑_*i*=1_^*m*^(*z*_*i*_^∗−^/*x*_*ik*_))/(1 + (1/*s*)∑_*r*=1_^*s*^(*z*_*r*_^∗+^/*y*_*rk*_)) = ρ*.

**Theorem 5 .**

If *DMU*_*k*_ is not efficient in model (2), that is, not SBM-efficient, the input excesses (*s*_*i*_^−^) and output shortfalls (*s*_*i*_^+^) identified by model (6) are the same as the those identified by model (2); in other words, *s*_*i*_^∗−^ = *z*_*i*_^∗−^, *s*_*r*_^∗+^ = *z*_*r*_^∗+^.

**Proof**

If DMU_*k*_ is evaluated by model (4) and is not efficient, then DMU_*k*_ ∈ *P*_*c*_′ and also *t*_*i*_^−∗^ = *t*_*r*_^+∗^ = 0. As a result, model (6) is degenerated to model (2) and then *s*_*i*_^∗−^ = *z*_*i*_^∗−^ and *s*_*r*_^∗+^ = *z*_*r*_^∗+^.

**Theorem 6 .**

According to the obtained amount of super efficiency φ*, three cases are identified as follows:

- If φ* > 1, φ* ≤ δ*;
- If φ* = 1, φ* = δ* = ρ*;
- If φ* < 1, φ* = ρ*.

**Proof**

(a) Since φ* > 1, DMU_*k*_ does not belong to *P*_*c*_′. Thus, according to Theorem 3,  φ* ≤ δ*.

(b) If φ* = 1, DMU_*k*_ is placed on *P*_*c*_′ frontier. This means that in model (5) the amount of *t*_*i*_^−∗^, *t*_*r*_^+∗^ = 0. Therefore, δ* = (1 + (1/*m*)∑_*i*=1_^*m*^(*t*_*i*_^−∗^ *x*_*i*_^max⁡^/*x*_*ik*_))/(1 − (1/*s*)∑_*r*=1_^*s*^(*t*_*r*_^+∗^ *y*_*r*_^max⁡^/*y*_*rk*_)) = 1. Moreover, φ* = (1 − (1/*m*)∑_*i*=1_^*m*^(*s*_*i*_^∗−^/*x*_*ik*_))/(1 + (1/*s*)∑_*r*=1_^*s*^(*s*_*r*_^∗+^/*y*_*rk*_)) = 1. Thus, considering the definition of *ρ* in model (2) and Theorem 3, φ* = δ* = ρ*.

(c) In the third case, in which φ* < 1, based on the mentioned definition of super efficiency, φ* = (1 − (1/*m*)∑_*i*=1_^*m*^(*s*_*i*_^∗−^/*x*_*ik*_))/(1 + (1/*s*)∑_*r*=1_^*s*^(*s*_*r*_^∗+^/*y*_*rk*_)). Besides, according to model (5), *t*_*i*_^−∗^ = *t*_*r*_^+∗^ = 0. Consequently,  δ* = (1 + (1/*m*)∑_*i*=1_^*m*^(*t*_*i*_^−∗^ *x*_*i*_^max⁡^/*x*_*ik*_))/(1 − (1/*s*)∑_*r*=1_^*s*^(*t*_*r*_^+∗^ *y*_*r*_^max⁡^/*y*_*rk*_)) = 1. Using model (5), it is shown that φ* = ρ*.

**Theorem 7 .**

Model (5) is unit invariant.

**Proof**

If in model (5) either all inputs or outputs are divided or multiplied by a number, the model is unit invariant and the optimal solution does not change because

(9) Min: δ=1+(1/m)∑i=1m(ti−(ximax⁡/K)/(xik/K))1−(1/s)∑r=1s(tr+(yrmax⁡/K′)/(yrk/K′))s.t.:  ∑j=1,j≠knλjxijK−ti−ximax⁡K≤xikK, ∀i   ∑j=1,j≠knλjyrjK′+tr+yrmax⁡K′≥  yrkK′, ∀r,       λj≥0, ti−≥0, tr+≥0.

After simplifying *K* of objective function and constraints, model (9) degenerates to model (5).

**Theorem 8 .**

The identified projection from model (6) is Pareto-efficient.

**Proof**

2 cases are considered for DMU_*k*_.

*Case 1*. DMU_*k*_ belongs to spanned production possibility set by DMU_*i*_(*i* ≠ *k*). In this case, model (6) degenerates to SBM model and the projection is Pareto-efficient.

*Case 2*. If DMU_*k*_ does not belong to production possibility set, then (*t*_*i*_^−∗^ *x*_*i*_^max⁡^, *t*_*r*_^+∗^ *y*_*r*_^max⁡^) is placed on frontier of PPS spanned by all DMU_*s*_ excluding DMU_*k*_. It is claimed that the point (*x*_*ik*_ + *t*_*i*_^−∗^ *x*_*i*_^max⁡^ − *s*_*i*_^∗−^, *y*_*rk*_ − *t*_*r*_^+∗^ *y*_*r*_^max⁡^ + *s*_*r*_^∗+^) is Pareto-efficient.

Proof by contradiction: if the above-mentioned point is not Pareto-efficient, then there exists a point like $\left(x_{ik}+t_{i}^{-*}x_{i}^{\max}-{\overset{-}{s}}_{i}^{-},y_{rk}-t_{r}^{+*}y_{r}^{\max}+{\overset{-}{s}}_{r}^{+}\right)$ which dominates (*x*_*ik*_ + *t*_*i*_^−∗^ *x*_*i*_^max⁡^ − *s*_*i*_^∗−^, *y*_*rk*_ − *t*_*r*_^+∗^ *y*_*r*_^max⁡^ + *s*_*r*_^∗+^). This contradicts the optimal property of *s*_*r*_^∗+^, *s*_*i*_^∗−^.

**Theorem 9 .**

Model (5) is always feasible.

**Proof**

If DMU_*k*_ ∈ *P*_*c*_′, according to definition of *P*_*c*_′,

(10) ∑j=1,j≠knλjxij≤xik,∑j=1,j≠knλjyrj≥yrk,∃  λ−, λ−≥0.

Choosing $(\overset{-}{\lambda }$,  *t*_*i*_^−^ = 0,  *t*_*r*_^+^ = 0), a feasible solution of model (5) can be obtained. Otherwise, if DMU_*k*_ ∉ *P*_*c*_′, the corresponding solution for that should be found. For this purpose, at first, we should suppose that $\overset{-}{\lambda }=1$. The input constraints of model (5) are ∑_*j*=1,*j*≠*k*_^*n*^ *x*_*ij*_ − *x*_*ik*_ ≤ *t*_*i*_^*t*^ *x*_*i*_^max⁡^. Since *x*_*i*_^max⁡^ > 0, *y*_*r*_^max⁡^ > 0, ∀*i*,  *r*, ${\overset{-}{t}}_{i}^{-}$ is defined as follows:

(11) $$\begin{array}{c} {\overset{-}{t}}_{i}^{\;-}=\{\begin{array}{cc} \frac{\sum_{j=1,j\neq k}^{n}x_{ij}-x_{ik}}{x_{i}^{\max}} & \overset{n}{\underset{j=1,j\neq k}{\sum }}x_{ij}>x_{ik}\\ 0 & \text{o}.\text{w}. \end{array} \end{array}$$

Moreover, corresponding to output constraints $\overset{-}{\lambda }=1$,  *y*_*r*_^max⁡^ > 0, ∀*r*, ${\overset{-}{t}}_{r}^{+}$ is defined as follows:

(12) $$\begin{array}{c} {\overset{-}{t}}_{r}^{+}=\{\begin{array}{cc} \frac{y_{rk}-\sum_{j=1,j\neq k}^{n}y_{rj}}{y_{r}^{\max}}\; & y_{rk}>\overset{n}{\underset{j=1,j\neq k}{\sum }}y_{rj}\\ 0 & \text{o}.\text{w}. \end{array} \end{array}$$

Considering $\left(\overset{-}{\lambda },{\overset{-}{t}}_{i}^{\;-},{\overset{-}{t}}_{r}^{+}\right)$, a feasible solution for model (5) is achieved.

Model (6) is a fractional programming model that can be modified to a linear programming as follows. That occurs by substituting variable *t* which itself is defined by 1/*t* = 1 + (1/*s*)∑_*i*=1_^*s*^(*s*_*r*_^+^/*y*_*rk*_)

(13) Min: t−1m∑i=1m(s^i −xik)s.t.:  1=t+1s∑r=1s(s^r +yrk),   ∑j=1,j≠knλ^jxij−tti∗ximax⁡+s^i −=txik, ∀i,   ∑j=1,j≠knλ^jyrj+ttr∗yrmax⁡−s^r +=tyrk, ∀r,           λ^j≥0, s^r≥0, s^i≥0.

In model (13), it should be considered that *t* > 0. The optimal solution for model (13) is $\left({\hat{\lambda }}_{j}^{*},{\hat{s}}_{i}^{\;*-},{\hat{s}}_{r}^{\;*+},t^{*}\right)$ by which the optimal solution of model (6) is obtained as follows:

(14) $$\begin{array}{c} \lambda_{j}^{*}=\frac{{\hat{\lambda }}_{j}^{\;*}}{t^{*}},\;\;s_{i}^{*-}=\frac{\;{\hat{s}}_{i}^{\;*-}}{t^{*}},\\ s_{r}^{*+}=\frac{{\hat{s}}_{r}^{\;*+}}{t^{*}}. \end{array}$$

## 4. Numerical Examples

Two examples of Tone [26, 46] were revisited and ranked through evaluation of units by both our and Tone's methods. In the first example, it is supposed that the 5 decision making units have 2 inputs and 2 outputs. The related data and results are listed in Table 1. The first column represents units. In columns 2 and 3, information about inputs is presented; however, that of outputs is listed in columns 4 and 5. The sixth column (ρ*) shows the amount of optimal solution for model (2). Column seven (δ*) represents optimal solution for each unit in model (4). The eighth column indicates the amounts of super efficiency which is obtained through the proposed method. For calculating φ*, first, the units are evaluated by model (5) and then by substituting point (*x*_*ik*_ + *t*_*i*_^−∗^ *x*_*i*_^max⁡^, *y*_*rk*_ − *t*_*r*_^+∗^ *y*_*r*_^max⁡^) in model (6) the optimal solution is obtained and considering the optimal amounts of (*t*_*i*_^−∗^, *t*_*r*_^+∗^), (*s*_*i*_^−∗^, *s*_*r*_^+∗^) and substituting them in the proposed definition of super efficiency *(∗)*, the amount of φ* is obtained. Finally, in column 9 the unit ranks are presented based on φ*.

As it is noticed in Table 2, despite the fact that the amount of φ* for unit 5 is bigger than that of unit 3, by calculating super efficiency through the proposed method, it is observed that the rank of unit 3 is better than that of unit 5.

In the second example, 6 decision making units with 4 inputs and 2 outputs are considered. In this example, the units are evaluated by both proposed method and Tone's method and then ranked. Table 2 shows the data of those units. As it is noticed, ρ* = 1. This means that all units are SBM-efficient and are located on the frontier. DMU_5_ in both methods has the first rank.

As it is seen, ranks of units in proposed method (φ*) were 4, 2, 5, 3, 1, and 6, respectively, while those of Tone's got 6, 2, 4, 3, 1, and 5, respectively.

## 5. Conclusion

In data envelopment analysis, a wide variety of models have been presented by using which decision making units can be evaluated and ranked, though most of them do not have properties such as feasibility in all cases, being unit invariant, ranking nonextreme efficient units, and finding strong Pareto-efficient projection for all units.

In this study, in order to calculate super efficiency of units and rank them, Anderson-Peterson's idea was utilized in two stages. In the first stage, the unit under evaluation was projected on production possibility set spanned by the rest of the DMUs and, in the second stage, the first projection point is transferred to a Pareto-efficient point. Then, by applying slack variables in the definition of super efficiency units were ranked. The introduced model in this paper has several advantages including having feasibility, obtaining a Pareto-efficient projection, and being unit invariant. The other advantage of the model is that it involves the amount of inefficiency in the amount of super efficiency and consequently affects ranking units in the case that the first projection point is placed on weak frontier. However, the problem of ranking nonextreme efficient units still remains. The proposed model is similar to SBM and as it was observed in previous sections, it is equivalent to Tone's model, though the obtained results are different due to the new definition of super efficiency.

## Figures and Tables

**Table 1:** 

DMU	Inp. 1	Inp. 2	Out. 1	Out. 2	ρ*	δ*	φ*	Rank
1	4	3	2	3	0.7980	1	0.7980	3
2	6	3	2	3	0.5682	1	0.5682	5
3	8	1	6	2	1	1.3333	1.3333	1
4	8	1	6	1	0.6667	1	0.6667	4
5	2	4	1	4	1	1.4545	0.9919	2

**Table 2:** 

DMU	Inp. 1	Inp. 2	Inp. 3	Inp. 4	Out. 1	Out. 2	ρ*	δ*	φ*	Rank
1	80	600	54	8	90	5	1	1.0116	0.7798	4
2	65	200	97	1	58	1	1	1.4146	0.9164	2
3	83	400	72	4	60	7	1	1.0781	0.7677	5
4	40	1000	75	7	80	10	1	1.1563	0.7927	3
5	52	600	20	3	72	8	1	1.5858	1.8107	1
6	94	700	36	5	96	6	1	1.0198	0.7590	6
